# Intrathecal Fentanyl With a Paracervical Block Is Safe and Effective for Elective Termination of Pregnancy in a Patient With Primary Pulmonary Hypertension

**DOI:** 10.7759/cureus.22699

**Published:** 2022-02-28

**Authors:** Joshua Younger, Mohamed Fayed, Gaurav Chauhan, Nicholas Mantel, Donald Penning

**Affiliations:** 1 Obstetric Anesthesia, Henry Ford Health System, Detroit, USA; 2 Anesthesiology, Pain Management and Perioperative Medicine, Henry Ford Health System, Detroit, USA; 3 Anesthesiology and Perioperative Medicine, University of Pittsburgh Medical Center Presbyterian, Pittsburgh, USA; 4 Anesthesiology and Perioperative Medicine, MedStar Harbor hospital, Baltimore, USA; 5 Anesthesiology, Wayne State University School of Medicine, Detroit, USA

**Keywords:** postoperative bleeding, intrathecal opioids, regional anesthesiology, induced abortion, dilatation and curettage, maternal and infant mortality, idiopathic pulmonary arterial hypertension, paracervical block, termination of pregnancy, pulmonary hypertension in pregnancy

## Abstract

Pulmonary hypertension (PH) in pregnancy, irrespective of etiology, is associated with significant maternal morbidity and mortality. This case describes a novel approach to providing anesthesia for a hemodynamically fragile patient. It demonstrates the careful planning and weighted decision-making that is required when approaching a parturient with severe pulmonary hypertension. The patient's previous pulmonary artery catheterization showed right ventricular systolic pressure of 78 mmHg and pulmonary artery pressure of 78/20 mmHg. The patient presented with worsening dyspnea and a decision was made to proceed with the termination of pregnancy via dilatation and curettage (D&C). Anesthesia was conducted with combined intrathecal fentanyl with a paracervical block using lidocaine 2%. The patient had a complication of post-procedure hemorrhage secondary to uterine atony that required careful monitoring and judicious use of uterotonic medications. A decision was made to use oxytocin due to its favorable effect profile compared to other uterotonic medications. We hope this anesthesia technique will aid in the future management of these challenging cases.

## Introduction

Pulmonary hypertension (PH) can be a primary entity or may result from several underlying systemic conditions. Pregnancy in patients with PH, irrespective of etiology, is associated with significant maternal morbidity and mortality ranging from 30% to 50% [1]. Physiological changes associated with pregnancy include an increase in cardiac output, a decrease in systemic vascular resistance as well a hypercoagulable state. These changes will lead to worsening pulmonary hypertension and right-sided heart failure as well. 

We here describe a parturient with a known history of severe idiopathic pulmonary hypertension who presented for elective termination of pregnancy through dilation and curettage (D&C). The patient had worsening dyspnea in this pregnancy and was recently listed for a heart and lung transplant. The patient underwent successful D&C utilizing only intrathecal fentanyl and a paracervical block with 2% lidocaine. This method allowed for stringent hemodynamic control while providing sufficient surgical anesthesia. However, the course was complicated by uterine atony with postpartum hemorrhage which was medically managed to temporize the situation but required thoughtful and vigilant administration given the hemodynamic fragility of this patient.

## Case presentation

A 22-year-old female presented for elective termination of pregnancy at 10 weeks gestational age.

The patient's past medical history was significant for primary pulmonary arterial hypertension. She underwent right heart catheterization four months prior to the presentation which reported a right ventricular pressure (RVP) of 78/20 mmHg, and pulmonary artery pressure (PAP) of 80/35 mmHg. Echocardiogram demonstrated a preserved left ventricular ejection fraction (EF) of 65% with evidence of right ventricular enlargement, tricuspid valve regurgitation and interventricular septum deviated towards left side. The patient was on three liters per min of home oxygen via nasal cannula as well as endothelin receptor antagonist (Bosentan), and phosphodiesterase inhibitor (Tadalafil). She was being considered for eventual heart and lung transplantation.

The patient reported worsening dyspnea for the past six weeks and symptoms consistent with New York Heart Association class III. Her oxygen saturation was 92% on three liters per minute of oxygen. The patient denied fever, cough, sputum production, rigors or shakes. The patient also denied chest pain or palpitations. Examination showed signs of right-sided heart failure with peripheral edema, congested neck veins and increased work of breathing. Transthoracic echocardiogram showed features of worsening right-sided heart functions. Rest of the workup including laboratory studies and electrocardiogram were within normal limits. A decision was made by her pulmonologist and obstetrician for elective termination of pregnancy. 

The patient presented to our facility and was reviewed by senior obstetric and cardiac anesthesiologists. Considering the patient's significant medical history of pulmonary hypertension, a decision was made to do a combined spinal anesthesia with intrathecal fentanyl with paracervical block with total of 10 ml of plain lidocaine 2%. Cardiac anesthesiologist was on standby and ready for transesophageal echocardiogram if any decompensation or extra monitoring was needed. 

Standard American Society of Anesthesia (ASA) monitors were applied including electrocardiogram, blood pressure, and pulse oximetry. Her ASA physical status was considered to be 4. A peripheral 18-gauge catheter and a radial arterial line were placed. A spinal anesthetic using 25 micrograms of fentanyl was instilled at the L3-4 interspace using a 27-gauge pencil-point needle. One mg of midazolam was administered intravenously (IV) for anxiolysis. A paracervical block was then preformed using 10 ml of 2% lidocaine as a supplement to spinal fentanyl to address pain from cervical dilation. During the Intra-operative course, her hemodynamics were stable with mean arterial blood pressure above 65 mmHg, and oxygen saturation above 93%. The patient didn't require any supplemental vasopressors or inotropes. She didn't feel pain from the D&C. 

Following the D&C, the patient had postoperative bleeding from uterine atony. The obstetrician vigorously massaged the uterus and requested a 10 units bolus of oxytocin followed by an infusion. A calculated risk/benefit assessment was made, and the authors elected for administering an oxytocin infusion in escalating dosage as opposed to a bolus method. The patient was stable throughout the 15-minute event and had an uneventful recovery. Estimated blood loss was less than 500 ml. 

## Discussion

Physiological changes of pregnancy can lead to high maternal morbidity and mortality in patients with PH [2,3]. Moreover, most of the drugs used for the optimal treatment of PH are contraindicated in pregnancy due to teratogenicity which limits the optimization of the patient [4]. The first trimester is widely regarded as the safest time for therapeutic abortion, as pregnancy-related plasma volume changes are less pronounced. The physiological parameters to be mindful of when choosing an anesthetic plan involve optimizing the systemic and right atrial pressures, maintaining a judicious fluid balance, and avoiding factors that may increase PAP [5-7] (see Table 1).

**Table 1:** Factors that increase and decrease pulmonary vascular resistance PVR: Pulmonary vascular resistance, FiO2: Inspired oxygen concentration, PaCO2: Arterial carbon dioxide tension, PEEP: Positive end-expiratory pressure

Factors that increase PVR	Factors that decrease PVR
Low FiO_2_	High FiO_2_
High PaCO_2_	Low PaCO_2_
Acidosis	Alkalosis
High airway pressure and PEEP	Spontaneous breathing and low PEEP
Hypothermia	Nitric oxide
Vasopressors	Vasodilator e.g., Nitroglycerine
Epinephrine	Dobutamine, milrinone

**Figure 1:**
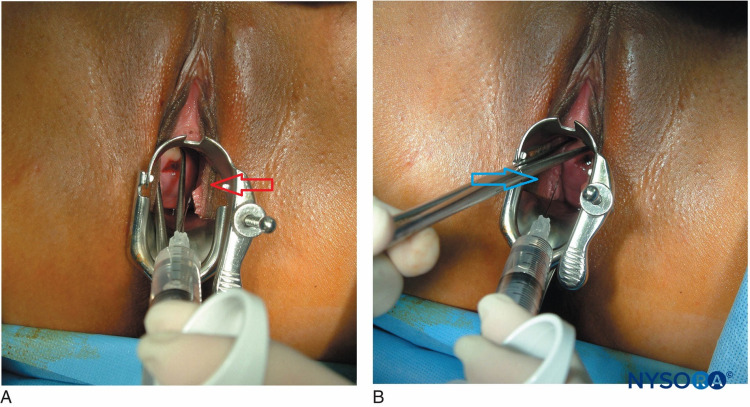
Paracervical block Red arrow: Left-sided paracervical block at 4 o'clock position, Blue arrow: Right-sided paracervical block at 8 o'clock position Source: NYSORA.COM, NYSORA grants the permission to download and use NYSORA’s teaching material [15].

During the perioperative period, in addition to standard ASA monitoring, invasive blood pressure monitoring and the ability for close monitoring of RV function is essential. A pulmonary artery catheter might be indicated, but there is no consensus regarding its routine use. In anticipation of hemodynamic compromise, intravenous prostaglandin (PG) along with the vasopressors and inotropic agents should be readily available [8]. General anesthesia (GA) has many drawbacks in patients with PH as it may be associated with a decrease in cardiac contractility, an increase in PVR and mean pulmonary arterial pressure (mPAP) during laryngoscopy and intubation or positive-pressure ventilation [9]. Consequently, there is a significantly increased risk of mortality in patients receiving GA as compared to regional anesthesia. Using large doses of local anesthetic (LA) in a single-shot spinal should be avoided as it may induce extensive sensory and motor block along with hypotension which may increase the risk of hemodynamic instability [10]. A titrated epidural anesthetic has traditionally been considered the best approach to regional anesthesia [11]. A low dose of LA via combined spinal-epidural anesthesia is also an attractive option as it provides a denser perineal sensory block than epidural anesthesia alone, with minimal risk of hypotension [11,12]. However, the use of an intrathecal opioid without any LA can provide excellent analgesia and is an apt technique for short procedures like a D&C. However, the advantage of using an opioid only spinal technique is that there is minimal concern for major hemodynamic derangement. Another opinion is to supplement paracervical block with IV short-acting opioids such as fentanyl rather than intrathecal opioids. However, this can come at the cost of systemic side effects of opioids, for example, respiratory depression. The literature has demonstrated that a paracervical block (Figure 1) [13] is insufficient as a sole anesthetic for a D&C [14]. The concern of using an opioid only spinal technique is that the analgesic may also be insufficient as the sole anesthetic. Given that the complications associated with a paracervical block are rare, the addition of a paracervical block can be used to supplement the opioid only spinal technique. 

Once uterine atony is diagnosed, uterine massage is initiated. Either during the massage or once the massage fails to yield the desired results, uterotonic agents should be considered. The first-line drug for uterine atony is oxytocin. The plasma half-life of oxytocin is one to six minutes, and the clinical response is within minutes with IV administration. The physician should be aware that oxytocin, especially the IV push, can cause hypotension via systemic vasodilation and can exacerbate PH by impeding right coronary blood flow leading to RV failure. Furthermore, the post-abortion uterus in the first and second trimester has fewer oxytocin receptors which might lead to a therapeutic failure or lower the efficacy of the drug [16]. Other alternatives to oxytocin that are commonly employed in the general population are ergot alkaloids and prostaglandins. In a setting of PH, ergot alkaloids are contraindicated in the parturient with primary pulmonary hypertension and can increase mPAP leading to decompensated RV failure [17] or coronary vasospasm [18]. Prostaglandins employed as uterotonic agents include carboprost and misoprostol. Carboprost has a similar pharmacodynamic effect on pulmonary vasculature as ergot alkaloids and is unsuitable for PH subjects [19]. Misoprostol is reported to have no cardiac effects and can be used as an alternative or in addition to oxytocin. However, its efficacy as a uterotonic agent is not well documented and limited to a few cases reports.

## Conclusions

Our observations reveal that spinal opioids in conjunction with a paracervical block are an effective and safe anesthetic approach during elective termination of pregnancy in a parturient with PH. Another alternative to spinal opioids is to consider titrated doses of IV short-acting opioids such as fentanyl with careful monitoring of respiratory status and systemic side effects. With patients who have PH, one should always anticipate adverse hemodynamic instability and have a therapeutic algorithm for potential complications. Regardless of recent medical advances and despite optimal strategies, the PH parturient may rapidly decompensate. The use of oxytocin infusion in escalating doses can safely terminate uterine atony during early pregnancy without adverse events on patients with PH. A perioperative therapeutic algorithm is vital for managing complications in patients with PH. A multi-disciplinary approach is indispensable for positive outcomes in subjects with PH.

## References

[1] MC RM, DU LJ (1964). Primary pulmonary hypertension in pregnancy. Obstet Gynecol Surv.

[2] Pieper PG, Hoendermis ES (2011). Pregnancy in women with pulmonary hypertension. Neth Heart J.

[3] Fayed M, Giska MA, Shievitz RC, Attali A, Younger J (2021). Emergent cesarean delivery in a patient with Freeman-Sheldon syndrome complicated by preeclampsia, acute pulmonary embolism, and pulmonary edema: a case report. Cureus.

[4] Duarte AG, Thomas S, Safdar Z, Torres F, Pacheco LD, Feldman J, deBoisblanc B (2013). Management of pulmonary arterial hypertension during pregnancy: a retrospective, multicenter experience. Chest.

[5] Jaïs X, Olsson KM, Barbera JA (2012). Pregnancy outcomes in pulmonary arterial hypertension in the modern management era. Eur Respir J.

[6] Bonnin M, Mercier FJ, Sitbon O (2005). Severe pulmonary hypertension during pregnancy: mode of delivery and anesthetic management of 15 consecutive cases. Anesthesiology.

[7] Bajwa SJ, Bajwa SK (2013). Anaesthetic challenges and management during pregnancy: strategies revisited. Anesth Essays Res.

[8] Regitz-Zagrosek V, Blomstrom Lundqvist C, Borghi C (2011). ESC Guidelines on the management of cardiovascular diseases during pregnancy: the Task Force on the Management of Cardiovascular Diseases during Pregnancy of the European Society of Cardiology (ESC). Eur Heart J.

[9] Hemnes AR, Kiely DG, Cockrill BA (2015). Statement on pregnancy in pulmonary hypertension from the Pulmonary Vascular Research Institute. Pulm Circ.

[10] Sorensen MB, Jacobsen E (1977). Pulmonary hemodynamics during induction of anesthesia. Anesthesiology.

[11] Duggan AB, Katz SG (2003). Combined spinal and epidural anaesthesia for caesarean section in a parturient with severe primary pulmonary hypertension. Anaesth Intensive Care.

[12] Smedstad KG, Cramb R, Morison DH (1994). Pulmonary hypertension and pregnancy: a series of eight cases. Can J Anaesth.

[13] Gautier P, Jew E, Deschner B, Santos AC (2007). Chapter 53. Obstetric regional anesthesia. NYSORA Textbook of Regional Anesthesia and Acute Pain Management.

[14] Jackson E, Kapp N (2020). Pain management for medical and surgical termination of pregnancy between 13 and 24 weeks of gestation: a systematic review. BJOG.

[15] (2022). Contact NYSORA - NYSORA | NYSORA. https://www.nysora.com/contact-nysora/.

[16] O'Connell K, Jones HE, Simon M, Saporta V, Paul M, Lichtenberg ES (2009). First-trimester surgical abortion practices: a survey of National Abortion Federation members. Contraception.

[17] Secher NJ, Arnsbo P, Wallin L (1978). Haemodynamic effects of oxytocin (syntocinon) and methyl ergometrine (methergin) on the systemic and pulmonary circulations of pregnant anaesthetized women. Acta Obstet Gynecol Scand.

[18] Fayed M, Buffington B, Ibrahim R, Attali AY, Younger J (2021). Methylergometrine-induced myocardial infarction in the setting of a cesarean delivery. Cureus.

[19] Barney OJ, Haughney RVM, Bilolikar A (2012). A case of pulmonary oedema secondary to carboprost. J Obstet Gynaecol.

